# Lost and Found: The Broken Tip of a Posterior Tibial Nerve Catheter

**DOI:** 10.1155/cria/6326787

**Published:** 2026-01-28

**Authors:** Sebnem Rumeli, Kaan Yavuz, Mustafa Azizoglu, Ender Gumusoglu, Nureddin Teker, Mesut Bakir

**Affiliations:** ^1^ Division of Pain Medicine, Department of Anesthesiology and Reanimation, Mersin University Faculty of Medicine, Mersin, 33240, Türkiye; ^2^ Department of Anesthesiology and Reanimation, Mersin University Faculty of Medicine, Mersin, 33240, Türkiye; ^3^ Department of Orthopedics and Traumatology, Mersin University Faculty of Medicine, Mersin, 33240, Türkiye

## Abstract

Catheter breakage is a rare complication of continuous peripheral nerve blocks. Retained fragments pose a significant technical challenge, particularly when they migrate from the insertion site. We report a 39‐year‐old woman with Fabry disease and chronic foot pain refractory to pharmacologic therapy who, after a successful diagnostic posterior tibial nerve block, received a continuous peripheral nerve catheter. She initially experienced excellent analgesia but, by Day 4, developed severe neuropathic symptoms; imaging showed proximal migration of a catheter fragment. Computed tomography and fluoroscopy guided surgical retrieval of the fragment was performed. This case highlights the need for early recognition of catheter‐related complications, precise localization with advanced imaging, and timely surgical intervention to reduce adverse outcomes from retained catheter fragments.

## 1. Introduction

Continuous peripheral nerve block is commonly used for postoperative pain management. Less commonly, it is used in selected patients for the treatment of chronic pain [1]. Regardless of the technique applied, complications such as bleeding, nerve injury, and systemic local anesthetic toxicity may occur. Rarely, trauma occurring during catheter insertion or removal, or excessive traction applied to catheter, may lead to complications such as catheter breakage [2]. The removal of foreign body fragments remaining in the body after a catheter breakage involves certain technical challenges. Among these challenges, catheter displacement is considered as one of the most critical factors complicating surgical intervention. Preoperative localization of the catheter fragment facilitates surgical intervention; however, this may necessitate the use of multiple imaging modalities, such as plain radiography, computed tomography (CT), magnetic resonance imaging (MRI), or real‐time fluoroscopy [3].

This case aims to highlight the challenges associated with the surgical removal of a peripheral nerve catheter fragment following spontaneous breakage of the catheter tip in the lower extremity.

## 2. Case Presentation

A 39‐year‐old woman with Fabry disease and a prior amputation at the second toe MTP joint presented with a 2‐year history of refractory right foot pain. Due to inadequate response to pharmacologic treatment (including tramadol, paracetamol, and dexketoprofen), a diagnostic posterior tibial nerve block was performed at the level of the right malleolus. After a positive response, a continuous catheter targeting the posterior tibial nerve was planned. With the patient supine and the right lower extremity externally rotated, the posterior tibial neurovascular bundle was identified in the transverse plane using a high‐frequency linear ultrasound probe. A continuous peripheral nerve catheter was inserted adjacent to the posterior tibial nerve using a (Braun) continuous peripheral nerve catheter set under ultrasound guidance. A test dose of 5 mL of 0.5% bupivacaine with 5 mL of normal saline was administered [4]. The patient reported immediate numbness in the plantar foot, and at 12 h postprocedure, she reported a 50% reduction in her pain. An infusion regimen of 5 mL of 0.5% bupivacaine with 5 mL of normal saline every 12 h was initiated. The procedure and initial drug administration were uneventful.

**FIGURE 1:**
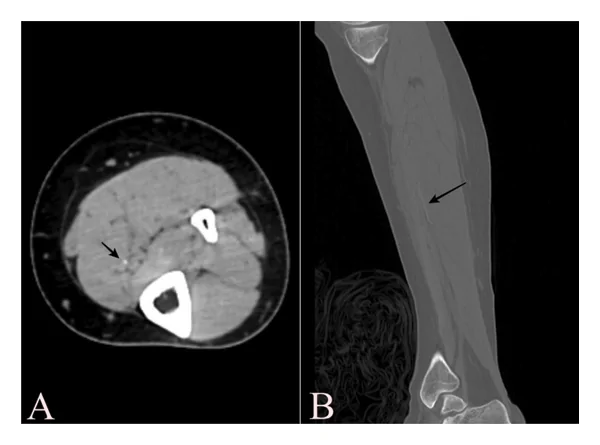
(A) Axial CT image demonstrating the catheter fragment at the mid‐cruris level. (B) Sagittal CT image showing the displaced catheter fragment extending proximally from the original insertion site.

Four days later, however, the analgesic efficacy was lost, and the patient developed new, severe radiating pain extending from the posterior thigh to the leg. Upon the patient’s request, the catheter was removed; however, it was noted that distal tip was missing. Initial evaluation with ultrasonography and plain radiography failed to identify the location of the retained fragment. Subsequent CT imaging revealed that the catheter tip had migrated proximally from the original insertion site (Figures 1A and 1B).

**FIGURE 2:**
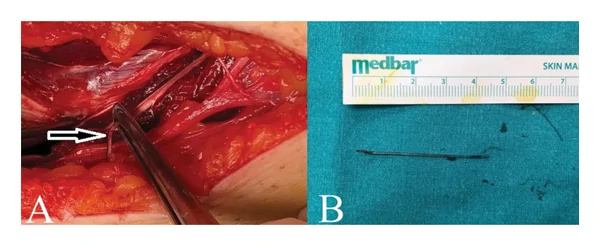
(A) Intraoperative view demonstrating the catheter fragment located between the muscle fascial planes. (B) Fragment of the peripheral nerve catheter following breakage and surgical removal.

The patient was referred to Orthopedics and scheduled for surgical exploration. On the day of surgery, the fragment’s location was confirmed and marked under fluoroscopic guidance. A targeted incision was made, and the catheter fragment was successfully retrieved from between the fascial planes (Figures 2A and 2B). Postoperatively, the patient’s new radiating neuropathic symptoms resolved. However, her underlying chronic foot pain persisted.

## 3. Discussion

The development of new‐onset neuropathic symptoms after peripheral nerve catheterization should raise suspicion for catheter‐related complications. In the present case, these symptoms were the primary reason for catheter removal. The pattern of severe radiating pain extending from the posterior thigh to the leg is consistent with the possibility that the migrated catheter tip lay adjacent to the sciatic nerve, most likely its tibial component. The complete resolution of symptoms after catheter fragment withdrawal supports this interpretation. Proximal migration of the catheter tip complicated both radiologic localization and surgical retrieval, underscoring the challenges of managing this rare but important problem.

Reported complication rates for peripheral nerve catheters range from 9% to 44%, with the most common issues being kinking, accidental dislodgement, occlusion, and leakage of local anesthetic [5]. Catheter breakage, by contrast, is far rarer but carries significant potential consequences. Most of the literature on breakage comes from thoracic and lumbar epidural catheters, where incidence has been reported between 0.002% and 0.04% [6]. Despite wider adoption of continuous peripheral nerve blocks, there remains a notable paucity of data on catheter breakage in peripheral nerve blocks—particularly in lower extremity applications—highlighting the need for further reporting.

Breakage is most often recognized during catheter removal or after related complications emerge. Excessive advancement appears to be a key contributor, frequently leading to knot formation. In one recent series, 11 of 20 patients had catheters advanced approximately 8 cm beyond the needle tip; several encountered resistance on removal, and subsequent evaluation revealed knotting [7]. Another mechanism is transection by the needle bevel during insertion [8]. In our case, the absence of technical difficulty during placement and the 4 days of effective analgesia make an intraprocedural transection unlikely. Instead, we suspect that the catheter’s proximity to the mobile ankle joint led to repeated kinking during flexion–extension, culminating in structural failure.

Retained peripheral nerve catheter fragments are generally considered inert, and conservative management is often recommended. However, despite this approach, case reports in the literature have documented a range of complications associated with retained catheter fragments. Khan et al. reported a case of a nonhealing ulcerative lesion in the sciatic nerve region that developed 5 months after peripheral nerve catheter placement, ultimately attributed to a retained catheter fragment [9]. In another report, surgical removal was performed in a patient presenting with persistent hip pain unresponsive to morphine treatment, where CT identified a 6‐cm catheter fragment in the femoral nerve region [9, 10]. In our patient, the emergence of new neuropathic symptoms prompted surgical retrieval, suggesting a direct and symptomatic link that necessitated intervention.

Detection is hampered by the catheter’s transparent, flexible material. In the literature, advanced radiological modalities such as CT and MRI have frequently been utilized to localize retained peripheral nerve catheter fragments. It is important to note, however, that not all catheter materials are MRI compatible, which may limit the applicability of this modality in certain cases [7, 11–13]. In our case, CT was chosen due to suspected proximal migration, and preoperative fluoroscopy was used to mark the fragment, enabling a targeted retrieval.

Anatomical location strongly influences removal complexity. Superficial fragments may be retrieved minimally invasively, whereas those embedded within muscle or fascial planes usually require more extensive approaches. Our fragment lay within the muscle fascia at a site distant from the original insertion, and its transparent nature, compounded by bleeding in the surgical field, severely hampered intraoperative visualization, directly contributing to the procedural challenge.

Catheter management in ambulatory patients may include telephone follow‐up, written instructions, and healthcare professional support when needed. Although no single approach has demonstrated clear superiority, literature suggests that the majority of patients are capable of safe home catheter management given proper instruction [14]. However, in the present case, drug administration performed by the patient at home may have delayed recognition of catheter migration. Clear and structured patient education focused on inspection of the insertion site, and recognition of early warning signs may help reduce the likelihood of such complications.

In conclusion, although peripheral nerve catheter breakage is uncommon, it should be suspected when analgesia is inadequate or new neurological symptoms arise. This case highlights that prompt evaluation using advanced imaging like CT is crucial for locating migrated fragments, and a collaborative, preoperatively planned surgical approach is essential for successful retrieval.

## Funding

No funding was received for this manuscript.

## Conflicts of Interest

The authors declare no conflicts of interest.
